# Rivaroxaban versus vitamin K antagonist treatment on the progression of coronary calcification: the IRIVASC-trial

**DOI:** 10.1038/s41598-024-67657-8

**Published:** 2024-07-30

**Authors:** Robert Stöhr, Sebastian Reinartz, Timm Dirrichs, Klaus Witte, Alexander Schuh, Vincent Brandenburg

**Affiliations:** 1 Department of Cardiology, RWTH University Hospital Aachen, Pauwelstrasse 30, 52074 Aachen, Germany; 2 Department of Cardiology and Angiology, University Hospital Frankfurt, Frankfurt, Germany; 3 Department of Radiology, University of Düsseldorf, Düsseldorf, Germany; 4 Department of Radiology, RWTH University Hospital Aachen, Aachen, Germany; 5 Department of Cardiology, St.-Katharinen-Hospital, Frechen, Germany; 6 Department of Cardiology and Nephrology, Rhein-Maas Klinikum, Würselen, Germany

**Keywords:** DOAC, Rivaroxaban, Vitamin K, Calcification, Cardiovascular biology, Calcification

## Abstract

Vitamin K antagonists (VKA) remain the only option of anticoagulation for people with mechanical valve replacement and due to their wider availability and lower acquisition costs, VKA’s remain widely used in low- and middle-income countries. It has been suggested that prolonged use of VKAs can increase the development of vascular and valvular calcification, though this effect has not been examined in larger randomized prospective trials. This investigator-initiated multicenter, prospective, randomized, open-label interventional trial randomized patients with baseline coronary or valvular calcification and an indication for prolonged oral anticoagulation therapy to Marcumar or Rivaroxaban. Patients were followed-up through repeat coronary computed tomographies to measure the progression of coronary and valvular calcification for up to 24 months. 192 patients were randomized between 2013 and 2018 to receive either Rivaroxaban or Marcumar and followed for up to 24 months. Coronary calcification significantly increased over time although there was no significant difference in progression between the groups after 12 and 24 months as measured by the Agatston score [360.7 (90.2; 1075.3) vs 380.4 (136.4; 1546.9) p = 0.69], the volume score [295.8 (93.0; 995.3) vs 335.5 (128.7; 1316.9) p = 0.95] and the mass score [58.5 (15.9; 172.0) vs 71.1 (24.8; 257.3) p = 0.5]. Dephosphorylated, uncarboxylated matrix Gla Protein (Dp-ucMGP) significantly decreased in the VKA group [Δ dp-uc MGP – 95.2 (− 554.1; 156.0) vs 231.3 (− 59.7; 388.1) p < 0.001]. There does not appear to be a relevant effect of vitamin K inhibition by the vitamin K antagonist marcumar upon coronary calcification.

## Introduction

Over the last 20 years direct oral anticoagulants (DOACs) have largely replaced vitamin K antagonists (VKA) for long-term oral anticoagulation therapy (OAT) in patients with atrial fibrillation or venous thromboembolism^1^. VKAs remain the only effective treatment option for people with mechanical valve replacements^2^ and due to their wider availability and lower acquisition costs, VKA’s remain widely used in low- and middle-income countries^3^.

Prolonged use of VKA has been postulated to accelerate cardiovascular calcification (CVC)^4^. Calcification induction is likely mediated through interference with the synthesis of anti-calcification vitamin K–dependent proteins, specifically through the interference with post-translational gamma-carboxylation of Matrix Gla Protein (MGP)^5^. MGP malfunction associates with premature cardiovascular calcifications^6,7^. Coronary artery calcification (CAC) has been shown to be closely related to the development of coronary artery disease. CAC as quantified by CT-scanning is nowadays even used as a screening marker to detect subclinical coronary artery disease^8^. Contrarily, animal and early human interventional data clearly suggest that the administration of vitamin K exerts anti-calcification effects and a decreased progression rate of CVC^9–12^.

The potential threat of VKA-treatment resulting in pro-calcific effects in the vasculature might have substantial impact for millions of patients for whom DOAC treatment is no guideline directed therapy or local availability is limited. Particularly in young patients, early VKA use might contribute to earlier ‘iatrogenic’ atherosclerotic disease or more rapid progression of the primary valve lesion. However, randomized controlled data on the association between oral anticoagulation therapy approaches and CVC development are sparse.

On this background, we designed the present study, the ‘Influence of rivaroxaban compared to vitamin K antagonist treatment upon development of cardiovascular calcification’ (IRIVASC study) (NCT02066662, registered 19.02.2014) specifically to explore whether VKA use was associated with greater progression of CVC than a non-vitamin K dependent oral anticoagulant (DOAC) in patients requiring long-term OAT.

## Methods

### Study design and approvals

IRIVASC was an investigator-initiated multicenter, prospective, randomized, open-label interventional trial conducted at 3 German hospitals. The trial protocol has been previously published^13^, was approved by the RWTH Aachen Ethics Committee (UK Aachen EC’ EK 239/12) and was registered with the Clinical Trials Database (https://www.clinicaltrials.gov; Unique identifier: NCT02066662). All study-related activities were carried out in accordance with the principles of the Declaration of Helsinki only following written and oral informed consent in each participant. The authors assume responsibility for the accuracy and completeness of the data and analyses, as well as for the fidelity of the trial and this report.

### Patients

We enrolled adults requiring long-term OAT according to current international guidelines for the treatment of atrial fibrillation (ACC/AHA/ESC-guidelines) and/or pulmonary embolism (ACCP/ESC guidelines) and being capable of taking either a DOAC *or* marcumar. Patients were included if a coronary or valvular calcification, or both exceeding an Agatston Score > 50 in at least one location as assessed by Mult-Slice-CT at screening was present. Exclusion criteria were intolerance of the imaging, an eGFR < 20 ml/min, serious life-limiting illness and cognitive dysfunction limiting compliance with study procedures. Patients in whom coronary artery calcification could not be reliably assessed including those with previous multi-vessel stents for coronary disease were also excluded^13^.

### Imaging

For calcium scoring, non-contrast ECG-triggered multi-slice CT (MSCT) of the whole heart was performed in a step-and-shoot-technique, as recommended by current guidelines^14^. All CT scanners employed in the present study were at least 64-slice MSCT scanners dedicated for cardiac imaging (SOMATOM Definition Flash, Siemens, Forchheim, Germany and Aquilion Prime, Canon Medical Systems, Neuss, Germany). To achieve reproducible and comparable calcium scoring results, every scanner are separately calibrated with a dedicated calcium calibration phantom (Anthropomorphic Cardio CT Phantom, size 300 × 200 × 100 mm, QRM Quality Assurance in Radiology and Medicine GmbH, Moehrendorf, Germany). The same basic scan parameters (80 mAs, 120 kV, 3/0.75 mm slice thickness, with the same rotation time, collimation, and pitch) were used at all sites after a scout view from the tracheal bifurcation to the bottom of the heart silhouette with adaption to participants’ physiques.

Imaging data were electronically transferred to a single dedicated post-processing and measurement workstation (SyngoCaScoring, Wizard; Siemens, Erlangen, Germany) for quantification of valvular and coronary calcification. Both valvular and coronary artery calcification (CAC) measurements were undertaken using dedicated software (Syngo MMWP, VA13A, Siemens, Erlangen, Germany). The volume score was determined by the calculated volume (Hounsfield units > 130 and a minimum size of 0.5 mm^3^) based on isotropic interpolation^15^. The Agatston score was calculated by multiplying density and size of the calcified areas as previously described^16^. In patients with single-vessel stenting, the stented area was manually excluded. In the event of poor quality data, or multiple stents, patients were excluded from further study participation. All measurements were performed by the same investigator with more than 10 years of experience in CAC scoring blinded to treatment randomization and the date of the examination. To improve intra-reader reproducibility, baseline and follow-up CT are presented to the reader immediately following each other in a random order.

### Intervention

Following baseline investigations, including baseline blood sampling and storage, patients were randomly assigned in a 1:1 ratio to either VKA (phenprocoumon Marcumar (R), titrated to target INR 2–3) or rivaroxaban (Xarelto) 20 mg once daily for patients with atrial fibrillation with eGFR > 49 ml/min and 15 mg once daily for patients with eGFR of 15 to 49 ml/min.

### Sample size and statistical analysis plan

The primary outcome was the progression of coronary and aortic valve calcification at 12 month. The follow-up time was prolonged to 24 month.

With a sample size of 95 per group (total population of 190), IRIVASC was powered to identify a difference in change of calcification of 96 units with a standard deviation of the change in volume score of 234 units between the groups, over 12 months with an 80% power using a type 1 error of 0.05 based on previous data measuring volume score change over 12 months in placebo treated patients with asymptomatic or mildly symptomatic aortic valve calcification^20^.

After confirming normality using a Shapiro–Wilk test, continuous baseline variables are presented as means (SD). Categorical variables are presented as number (%).

Log transformed outcome measurements were modeled using linear mixed models with fixed treatment effect (2 categories), time, log transformed baseline measurement, treatment-time interaction, and random intercepts grouped by subjects. Treatment effect was tested with an F-test using Kenward–Roger adjustment of the degrees of freedom. Outcome measurements at different time points (12 and 24 months) were compared separately in the two intervention groups using Kruskal–Wallis test.

## Results

### Patient characteristics

In total 192 patients (median age 70, 72% male, 96% atrial fibrillation) with a baseline Agatston Score > 50 at the coronary or valvular level were randomized to receive either Rivaroxaban (n = 96) or VKA (phenprocumon, Marcumar) (n = 96) and followed for up to 24 months. The baseline clinical characteristics including age, sex, past history and rates of diabetes mellitus along with calcification scores previously published and were comparable between the 2 groups^13^. For both indications atrial fibrillation and pulmonary embolism the INR target range was 2–3.

**Figure 1:**
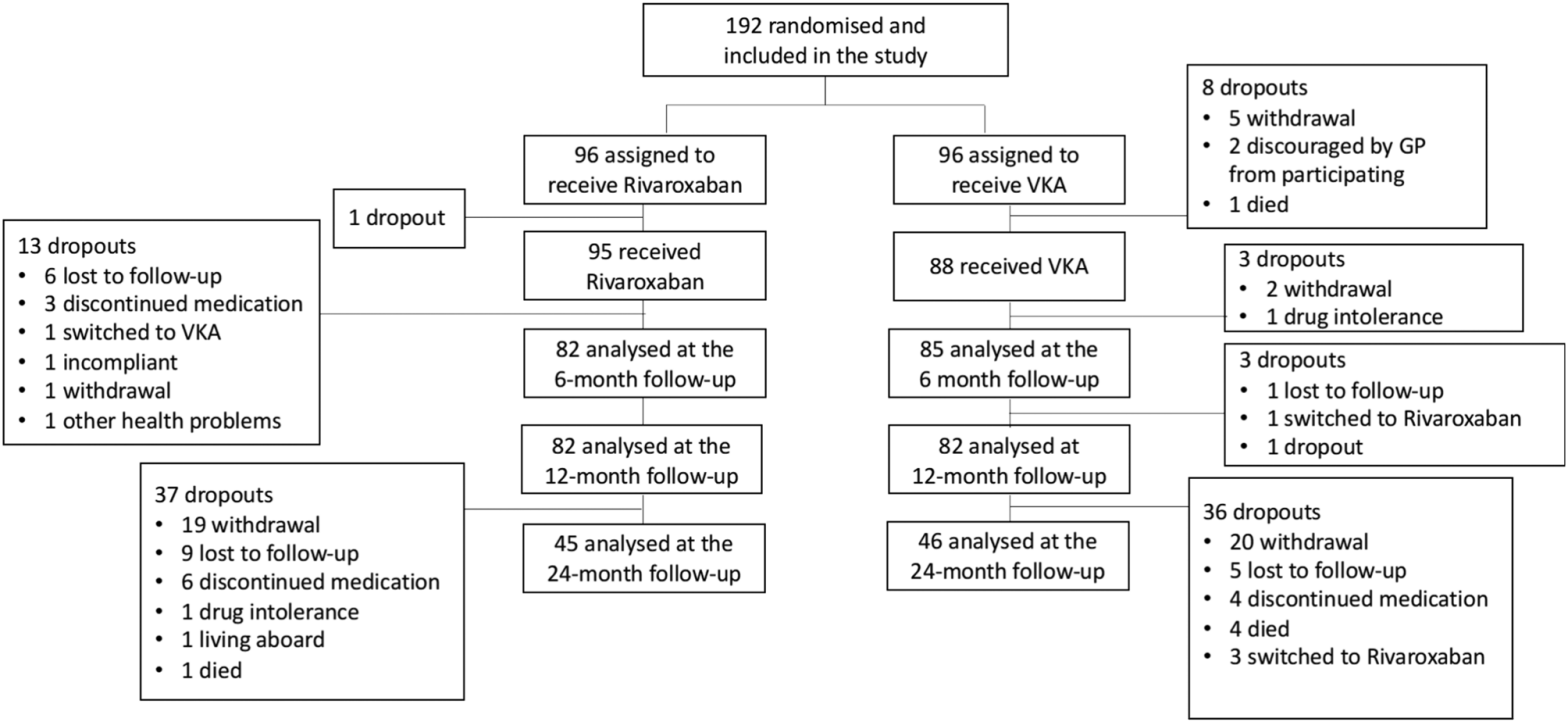
Patient flow chart.

After 12 months, coronary calcification follow-up data were available for 164 patients (82 in the DOAC group and 82 in the VKA group) while at 2 years, CT-data were available for 91 patients (45 in the DOAC group and 46 in the VKA group) (Fig. 1).

### Outcome

**Figure 2:**
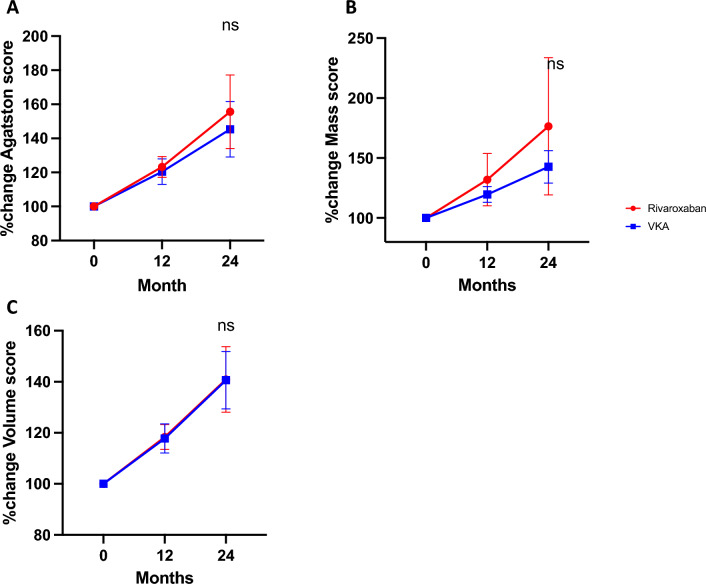
Percentage change of Agatston Score (**A**), Mass Score (**B**) and Volume Score (**C**) over the two year follow-up. Data are shown as Mean with 95% CI. (ns non significant).

There was no significant difference in the coronary calcifications scores between the groups at baseline (Table 1). At 12 months, coronary calcification was significantly greater in both groups, although there was no significant difference in progression between the groups after 12 and 24 months as measured by the Agatston score [360.7 (90.2; 1075.3) vs 380.4 (136.4; 1546.9) p = 0.69], the volume score [295.8 (93.0; 995.3) vs 335.5 (128.7; 1316.9) p = 0.95] and the mass score [58.5 (15.9; 172.0) vs 71.1 (24.8; 257.3) p = 0.5] (Table 1 and Fig. 2A–C).

**Table 1:** Calcification parameters over two year follow up.

Coronary Calcification	Rivaroxaban group							VKA group							*P *value
	t0 (n = 92)		t12 (n = 84)		t24 (n = 45)		*P* value	t0 (n = 87)		t12 (n = 82)		t24 (n = 46)		P value	
	Median	Q1; Q3	Median	Q1; Q3	Median	Q1; Q3		Median	Q1; Q3	Median	Q1; Q3	Median	Q1; Q3		
Coronary Agatston score	268.0	92.3; 988.4	307.3	89.1; 1200.8	360.7	90.2; 1075.3	0.59	328.1	131.5; 1587.7	353.6	170.5; 1778.4	380.4	136.4; 1546.9	0.71	0.69
Coronary volume score (ml)	247.7	90.4; 873.2	275.4	93.7; 1033.0	295.8	93.0; 995.3	0.66	292.3	127.4; 1322.1	333.6	155.1; 1481.9	335.5	128.7; 1316.9	0.70	0.95
Coronary mass score (mg)	43.7	16.6; 150.0	51.8	17.1; 196.3	58.5	15.9; 172.0	0.53	58.4	23.4; 274.7	62.5	30.1; 305.4	71.1	24.8; 257.3	0.69	0.50
ΔCoronary Agatston score			41.6	7.4; 114.9	77.2	29.9; 187.6	0.02			32.2	7.0; 136.6	104.1	36.1; 266.7	< 0.01	
Δ Coronary volume score (ml)			32.7	5.9; 85.3	61.8	22.9; 137.5	0.03			29.8	10.0; 102.1	87.6	30.8; 223.2	< 0.01	
Δ Coronary mass score (mg)			5.9	1.3; 19.3	14.9	4.0; 37.2	0.02			6.4	2.1; 21.5	18.1	6.5; 35.5	< 0.01	

MGP levels	Rivaroxaban group							VKA group							*P *value
	t0 (n = 85)		t12 (n = 72)		t24 (n = 45)		*P *value	t0 (n = 82)		t12 (n = 76)		t24 (n = 46)		P value	
	Median	Q1; Q3	Median	Q1; Q3	Median	Q1; Q3		Median	Q1; Q3	Median	Q1; Q3	Median	Q1; Q3		
dp-ucMGP	865.40	577.2; 1448.1	749.7	591.5; 1001.6	788.0	655.5; 948.5	0.45	1289.7	1010.7; 1696.4	1415.1	1108.8; 1717.0	1375.0	1152.0; 1933.0	0.25	< 0.001
Δ dp-uc MGP			− 37.9	− 594.3; 151.7	− 95.2	− 554.1; 156.0	0.90			103.7	− 123.6; 347.5	231.3	− 59.7; 388.1	0.29

### Biochemical measurements

**Figure 3:**
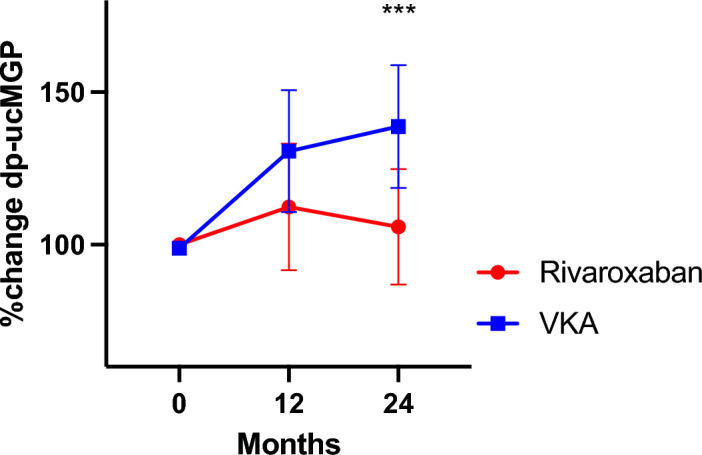
Percentage change of dp-ucMGP over the 2 year follow up. Data are shown as Mean with 95% CI. (***p < 0.001).

Treatment with the VKA antagonist resulted in significant increase of dp-ucMGP at 12 and 24 months compared to DOAC [Δ dp-uc MGP – 95.2 (− 554.1; 156.0) vs 231.3 (− 59.7; 388.1) p < 0.001] (Table 1 and Fig. 3).

## Discussion

IRIVASC is the first multicenter trial to prospectively compare the cardiovascular calcification effects of VKA and Rivaroxaban (DOAC) on the progression of coronary and aortic valve calcification over 24 months. Previous studies had raised concerns about an acceleration and premature occurrence of CVC in patients treated with VKA. In a cross-sectional analysis of standard CTC imaging, Bob et al*.* described higher levels of coronary artery calcification in patients using VKA^4^. Using serial intravascular ultrasound (IVUS) Andrews et al*.* noted that while VKA treatment did not promote plaque formation, it did encourage the development of plaque calcification^17^. Subsequent to these two observations, there have been only two further small prospective studies. Both proposed slower coronary plaque progression in patients treated with the DOACs rivaroxaban and apixaban compared with patients treated with the VKA warfarin^18,19^. The studies enrolled 97 and 56 patients respectively. However, the changes noted were modest in degree, requiring multiple adjustments despite randomization. Furthermore, their trials only reported a one year follow rather than the up to 2 years we are reporting in our study. Inconclusive results regarding VKA usage and premature calcification have been shown in 2 studies in dialysis patients^20,21^. However, generalizability of trial results in dialysis patients is limited due to their unique pro-calcific environment. Hence, the IRIVASC trial adds important novel insights into the field due to study size, population and follow-up time.

Our data provide additional insight around the results of a recent randomized, placebo-controlled study of vitamin K supplementation in 400 people with asymptomatic aortic stenosis^22^. After 2 years, there was no difference in the rate of progression of the aortic valve calcification between the groups. While in this study, the authors explored the effect of *supplementation* of Vitamin K2 on the progression of aortic valve calcification, our study examined the iatrogenic *reduction* of vitamin K through the use of the vitamin K antagonist warfarin. Taking these two studies together, despite the proposed molecular pathways, there does not appear to be a clinically relevant effect of vitamin K modulation on the progression of vascular calcification.

This finding is important for the future management of patients with valvular heart disease, in whom DOACs have shown insufficient efficacy^23^ and also for people living in low and middle-income countries where vitamin K antagonist therapy is frequently used for decades. Although data about life-long VKA application and potential effects upon cardiovascular health effects are still missing, IRIVASC does not raise safety concerns regarding intermediate-term progression of CAC.

Our clinical findings seem to contrast with the commonly described VKA-mediated increased calcification though the decrease in dp-ucMGP. This is confirmed by the alteration in the levels of the precursor to vitamin K-dependent Matrix Gla Protein (MGP) which demonstrates the expected interference with the availability of the calcification-inhibitory system MGP. However, it seems that this downregulation of active MGP in the circulation does not translate to a faster progression of CVC in our specific patient cohort. This disconnect between the biochemical alterations and the clinical outcomes was also noted in the vitamin K supplementation study described above^24^. These findings and the recent failure of denosumab and alendronic acid, both effectors of bone turnover and used to treat osteoporosis, to slow valvular calcification^25^, suggest that the effect of vitamin K and the Matrix Gla Protein pathway on the progression of valvular and coronary artery calcification appear more complex and might not be the key clinical drivers. Whilst others have proposed that progression might be dose dependent^26^, the doses required to achieve this might limit its clinical applicability to specific subgroups and particularly high risk. On the other hand, whether vitamin K antagonism at higher levels than for formal anticoagulation might be effective on cardiovascular calcification is likely to be clinically irrelevant.

We must stress, that our study only evaluated the effect of VKA therapy on the development of CVC rather the development of plaque burden itself. As such Beyer et al. were able to show that VKA administration in patients with AF led to an increase in plaque burden compared to DOAC^27^ therapy suggesting the effect may be related to other factors beside VKA’s ability to interfere with vascular calcification.

## Limitations

There are several limitations to highlight. Firstly, coronary or valvular calcification was an inclusion criterion such that IRIVASC could be considered a secondary prevention trial, perhaps limiting the relevance to the general population. Secondly, a large proportion of patients dropped out following the prespecified primary timepoint of 12 months scan, such that the study was underpowered at the time of the 24 months. The findings at 24 months should therefore be considered as exploratory. Finally, we did not record previous VKA usage or the time in treatment range for patients treated by VKA.

## Conclusions

In conclusion, there does not appear to be a relevant effect of vitamin K inhibition by the vitamin K antagonist phenprocoumon (Marcumar®) upon coronary or aortic valve calcification over 24 months of treatment. The rate of calcification progression was similar to patients treated with rivaroxaban. Hence, the data do not support the long-held belief the VKA-induced accelerated progression of cardiovascular calcification is mediated by decreased MGP activity in humans.

## Data Availability

The raw data that support the findings of this study are available from the Clinical Study Center (KKS) of the Clinic for Cardiology, Angiology and Intensive Care Medicine and the Clinic for Pneumology and Intensive Care Medicine of RWTH Aachen University Hospital, but restrictions apply to the availability of these data, which were used under license for the current study, and so are not publicly available. Data are however available from the authors upon request and with permission of KKS.

## References

[1] Hindricks, G. *et al.* 2020 ESC Guidelines for the diagnosis and management of atrial fibrillation developed in collaboration with the European Association for Cardio-Thoracic Surgery (EACTS): The Task Force for the diagnosis and management of atrial fibrillation of the European Society of Cardiology (ESC) Developed with the special contribution of the European Heart Rhythm Association (EHRA) of the ESC. *Eur. Heart J.* **42**, 373–498 (2021). 32860505 10.1093/eurheartj/ehaa612

[2] Eikelboom, J. W. *et al.* Dabigatran versus warfarin in patients with mechanical heart valves. *N. Engl. J. Med.* **369**, 1206–1214 (2013). 23991661 10.1056/NEJMoa1300615

[3] Ramakumar, V., Benz, A. P. & Karthikeyan, G. Long-term oral anticoagulation for atrial fibrillation in low and middle income countries. *Indian Heart J.* **73**, 244–248 (2021). 33865530 10.1016/j.ihj.2021.02.003 PMC8065364

[4] Weijs, B. *et al.* Patients using vitamin K antagonists show increased levels of coronary calcification: An observational study in low-risk atrial fibrillation patients. *Eur. Heart J.* **32**, 2555–2562 (2011). 21775389 10.1093/eurheartj/ehr226

[5] Kosciuszek, N. D., Kalta, D., Singh, M. & Savinova, O. V. Vitamin K antagonists and cardiovascular calcification: A systematic review and meta-analysis. *Front. Cardiovasc. Med.* **9**, 938567 (2022). 36061545 10.3389/fcvm.2022.938567 PMC9437425

[6] Koos, R. *et al.* Relation of oral anticoagulation to cardiac valvular and coronary calcium assessed by multislice spiral computed tomography. *Am. J. Cardiol.* **96**, 747–749 (2005). 16169351 10.1016/j.amjcard.2005.05.014

[7] Schurgers, L. J., Aebert, H., Vermeer, C., Bultmann, B. & Janzen, J. Oral anticoagulant treatment: Friend or foe in cardiovascular disease?. *Blood* **104**, 3231–3232 (2004). 15265793 10.1182/blood-2004-04-1277

[8] Cheong, B. Y. C. *et al.* Coronary artery calcium scoring: An evidence-based guide for primary care physicians. *J. Intern. Med.* **289**, 309–324 (2021). 33016506 10.1111/joim.13176

[9] Kremer, D. *et al.* Kidney function-dependence of vitamin K-status parameters: Results from the transplant lines biobank and cohort studies. *Nutrients* **13**, 3069 (2021). 34578950 10.3390/nu13093069 PMC8467091

[10] Nigwekar, S. U., Thadhani, R. & Brandenburg, V. M. Calciphylaxis. *N. Engl. J. Med.* **378**, 1704–1714 (2018). 29719190 10.1056/NEJMra1505292

[11] Brandenburg, V. M. *et al.* Slower progress of aortic valve calcification with vitamin K supplementation: Results from a prospective interventional proof-of-concept study. *Circulation* **135**, 2081–2083 (2017). 28533322 10.1161/CIRCULATIONAHA.116.027011

[12] Brandenburg, V. M. *et al.* Prevention of vasculopathy by vitamin K supplementation: Can we turn fiction into fact?. *Atherosclerosis* **240**, 10–16 (2015). 25744701 10.1016/j.atherosclerosis.2015.02.040

[13] Stohr, R. *et al.* Influence of rivaroxaban compared to vitamin K antagonist treatment upon development of cardiovascular calcification in patients with atrial fibrillation and/or pulmonary embolism. *Clin. Cardiol.* **45**, 352–358 (2022). 35332571 10.1002/clc.23819 PMC9019879

[14] Halliburton, S. S. *et al.* SCCT guidelines on radiation dose and dose-optimization strategies in cardiovascular CT. *J. Cardiovasc. Comput. Tomogr.* **5**, 198–224 (2011). 21723512 10.1016/j.jcct.2011.06.001 PMC3391026

[15] Callister, T. Q. *et al.* Coronary artery disease: Improved reproducibility of calcium scoring with an electron-beam CT volumetric method. *Radiology* **208**, 807–814 (1998). 9722864 10.1148/radiology.208.3.9722864

[16] Agatston, A. S. *et al.* Quantification of coronary artery calcium using ultrafast computed tomography. *J. Am. Coll. Cardiol.* **15**, 827–832 (1990). 2407762 10.1016/0735-1097(90)90282-T

[17] Andrews, J. *et al.* Warfarin use is associated with progressive coronary arterial calcification: Insights from serial intravascular ultrasound. *JACC Cardiovasc. Imaging* **11**, 1315–1323 (2018). 28734922 10.1016/j.jcmg.2017.04.010

[18] Win, T. T. *et al.* Apixaban versus warfarin in evaluation of progression of atherosclerotic and calcified plaques (prospective randomized trial). *Am. Heart J.* **212**, 129–133 (2019). 31002997 10.1016/j.ahj.2019.02.014

[19] Lee, J. *et al.* Randomized trial of rivaroxaban versus warfarin in the evaluation of progression of coronary atherosclerosis. *Am. Heart J.* **206**, 127–130 (2018). 30227941 10.1016/j.ahj.2018.08.007

[20] De Vriese, A. S. *et al.* Multicenter randomized controlled trial of vitamin K antagonist replacement by rivaroxaban with or without vitamin K2 in hemodialysis patients with atrial fibrillation: the valkyrie study. *J. Am. Soc. Nephrol.* **31**, 186–196 (2020). 31704740 10.1681/ASN.2019060579 PMC6935010

[21] Krueger, T. *et al.* Vitamin K1 to slow vascular calcification in haemodialysis patients (VitaVasK trial): A rationale and study protocol. *Nephrol. Dial. Transplant.* **29**, 1633–1638 (2014). 24285427 10.1093/ndt/gft459

[22] Diederichsen, A. C. P. *et al.* Vitamin K2 and D in patients with aortic valve calcification: A randomized double-blinded clinical trial. *Circulation* **145**, 1387–1397 (2022). 35465686 10.1161/CIRCULATIONAHA.121.057008 PMC9047644

[23] Connolly, S. J. *et al.* Rivaroxaban in rheumatic heart disease-associated atrial fibrillation. *N. Engl. J. Med.* **387**, 978–988 (2022). 36036525 10.1056/NEJMoa2209051

[24] Diederichsen, A. C. P., Hasific, S. & Dahl, J. S. Response by Diederichsen et al. to letter regarding article, “vitamin K2 and D in patients with aortic valve calcification: a randomized double-blinded clinical trial”. *Circulation* **146**, e227–e228 (2022). 36251786 10.1161/CIRCULATIONAHA.122.061691

[25] Pawade, T. A. *et al.* Effect of denosumab or alendronic acid on the progression of aortic stenosis: A double-blind randomized controlled trial. *Circulation* **143**, 2418–2427 (2021). 33913339 10.1161/CIRCULATIONAHA.121.053708 PMC8212878

[26] Saritas, T. *et al.* Vitamin K1 and progression of cardiovascular calcifications in hemodialysis patients: The VitaVasK randomized controlled trial. *Clin. Kidney J.* **15**, 2300–2311 (2022). 37216675 10.1093/ckj/sfac184 PMC9664584

[27] Beyer, C. *et al.* Relationship of anticoagulant therapies on coronary plaque progression: A longitudinal CTA analysis. *JACC Cardiovasc. Imaging* **13**, 169–170 (2020). 31492644 10.1016/j.jcmg.2019.07.021

